# Strategies for specific multimodal imaging of cancer-associated fibroblasts and applications in theranostics of cancer

**DOI:** 10.1016/j.mtbio.2024.101420

**Published:** 2024-12-24

**Authors:** Li Wen, Chengxue He, Yanhui Guo, Nina Zhou, Xiangxi Meng, Yuwen Chen, Cheng Ma, Hua Zhu, Zhi Yang, Lei Xia

**Affiliations:** a Key Laboratory of Carcinogenesis and Translational Research (Ministry of Education/Beijing), NMPA Key Laboratory for Research and Evaluation of Radiopharmaceuticals (National Medical Products Administration), Department of Nuclear Medicine, Peking University Cancer Hospital & Institute, Beijing, 100142, China; b Department of Nuclear Medicine, Union Hospital, Tongji Medical College, Huazhong University of Science and Technology, Hubei Key Laboratory of Molecular Imaging, Key Laboratory of Biological Targeted Therapy, The Ministry of Education, Wuhan, 430022, China; c Department of Radiology, Peking University Third Hospital, Beijing, 100088, China; d Department of Electronic Engineering, Beijing National Research Center for Information Science and Technology, Tsinghua University, Beijing, 100084, China; e Institute for Intelligent Healthcare, Tsinghua University, Beijing, 100084, China

**Keywords:** Fibroblast activating protein, Melanin nanoparticles, Multimodal imaging, Theranostics, Radioisotope therapy

## Abstract

Fibroblast activating protein (FAP) is up-regulated in cancer-associated fibroblasts (CAFs) of more than 90 % of tumor microenvironment and also highly expressed on the surface of multiple tumor cells like glioblastoma, which can be used as a specific target for tumor diagnosis and treatment. At present, small-molecule radiotracer targeting FAP with high specificity exhibit limited functionality, which hinders the integration of theranostics as well as multifunctionality. In this work, we have engineered a multifunctional nanoplatform utilizing organic melanin nanoparticles that specifically targets FAP, facilitating both multimodal imaging and synergistic therapeutic applications. This nanoplatform can perform positron emission tomography (PET), magnetic resonance imaging (MRI) and photoacoustic imaging (PAI) with strong near infrared absorption and metal chelating ability, achieving efficiently targeting accumulation and display long retention in the tumor region. Meanwhile, ^131^I-labeled nanoplatform for targeted radioisotope therapy (TRT) and photothermal therapy (PTT) were significantly suppressed tumor growth in glioblastoma xenograft models without obvious side effects. These results demonstrated that this novel nanoparticles-based theranostics nanoplatform can effectively enhance multimodal imaging and targeted radionuclide-photothermal synergistic therapy for solid tumors with FAP expression.

## Graphical abstract

Our study introduces unique strategy to enhance multimodal imaging and targeted radionuclide-photothermal synergistic therapy of solid tumor tumors by developing a multifunctional nanoplatform utilizing organic melanin nanoparticles that specifically targets fibroblast activating protein (FAP). Currently, small-molecule radiotracer targeting FAP with high specificity exhibit limited functionality, which hinders the integration of theranostics as well as multifunctionality. We used the high-loading function of melanin nanoparticles to label positron nuclide ^64^Cu, paramagnetic metal particle Mn^2+^ and label therapeutic nuclide ^131^I, together with the optical function function of melanin nanoparticles. This nanoplatform can perform positron emission tomography (PET), magnetic resonance imaging (MRI) and photoacoustic imaging (PAI) with strong near infrared absorption and metal chelating ability, achieving efficiently targeting accumulation and display long retention in the tumor region. Most importantly, ^131^I-labeled nanoplatform for targeted radioisotope therapy (RIT) and photothermal therapy (PTT) were significantly suppressed tumor growth in glioblastoma xenograft models without obvious side effects. This innovative strategy capitalizes the excellent biodegradability and biocompatibility of melanin nanoplatforms to provide a promising solution for tumour diagnosis and treatment, while ensuring a high degree of biosafety and in vivo elimination. By combining the advantages of targeted radionuclide-photothermal synergistic therapy, it has the potential to improve treatment outcomes and expanding the clinical utility of RIT/PTT protocols.Scheme 1. Schematic illustration of the theranostic nanoplatform FAPI-PEG-MNs for PET/MR/PA multimodal imaging and radioisotope-photothermal synergistic therapy of glioblastoma.

**Figure:**
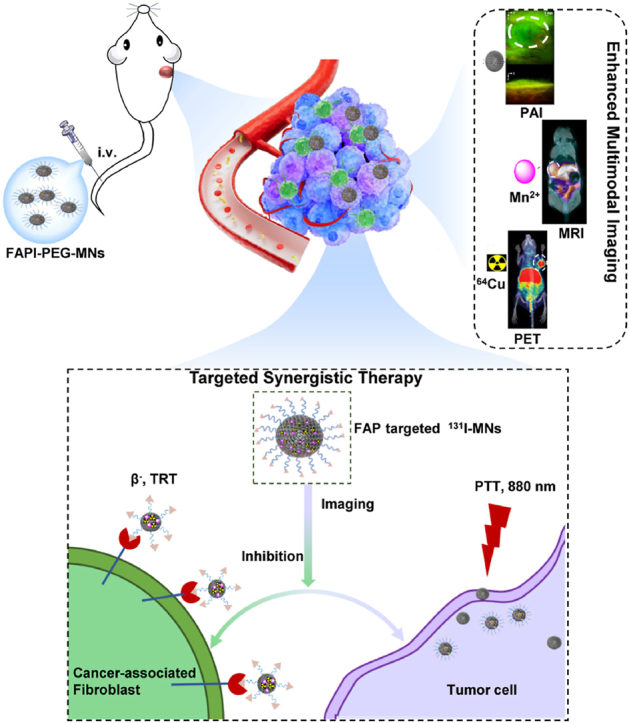

## 1 Introduction

Fibroblasts activating protein (FAP), a type II membrane-bound glycoprotein, is up-regulated in tumor-associated fibroblasts in more than 90 % of epithelial tumor and malignancies such as glioblastoma [1] and lung cancer [2]. It plays a key role in tumorigenesis, invasion, and metastasis, and is considered a potential target for tumor diagnosis and treatment due to its low or no expression in healthy tissues [3,4]. Although the applications of FAP detection in vivo, such as puncture, biopsy, and molecular imaging, are relatively mature at present, they all have some limitations and their application scope is also limited, but nuclear medicine imaging technology can monitor the expression of receptor proteins inside tumors in real time, providing patients with essential information for personalized and precise treatment [5]. Fibroblast activation protein inhibitor (FAPI) is a high affinity drug targeting FAP. Since the American Society of Nuclear Medicine highlighted in 2019 that Heidelberg University in Germany had completed the imaging research of [^68^Ga]Ga-FAPI-04 in fifteen types of tumors [6], FAP inhibitors have been extensively studied in molecular imaging and targeted therapy in nuclear medicine, including basic and clinical research. In 2012, Ballal et al. [7] first used [^68^Ga]Ga-DOTA-FAPI to perform PET/CT imaging on a breast cancer patient with systemic metastasis. Additionally, [^177^Lu] Lu-DOTA-FAPI was used for "compassionate" therapy on the patient, and achieved certain result. A series of compared studies showed that ^68^Ga FAPI had higher uptake in various primary and metastatic lesions and was more effective than ^18^F-FDG [[8], [9], [10]]. Therefore, it seems feasible to construct molecular probes targeting FAP for direct tumor radionuclide diagnosis and therapy. However, related clinical trials have not shown the expected revolutionary effects, and the shorten retention time of drugs in tumors may reduce their application in treatment.

With the rapid development of advanced nanotechnology, nanoplatform provide a new promising opportunity for diagnosis and therapy of solid tumor [11,12]. Nanoparticles, with their inherent advantages, can greatly improve their targeting and retention time of tumors by modification, indicating to develop multifunctional theranostics nanoprobe may provide a promising avenue for advancing FAP targeting diagnosis and therapy in the future. However, most nanomaterials are difficult to degrade in vivo and have poor tissue selectivity so far. In contrast, ultrasmall melanin nanoparticles (MNs) as novel endogenous organic nanoparticles with excellent biocompatibility, biodegradability, metal chelation ability, photothermal effect, and rich functional groups to enable labeling of multiple nuclides [13,14]. MNs can be prepared from natural melanin or dopamine by a simple and effective strategy with controllable particle size and stable structure [15,16]. In previous reports, MNs can be developed into multimodality imaging nanoprobes through radiolabeling and chelation of Fe^3+^, Gd^3+^, Mn^2+^ [[14], [17], [18], [19]], Moreover, they exhibit a monotonic broadband absorption profile across the ultraviolet–visible (UV–Vis) region, rendering its great potential for PA contrast agents and PTT therapy [14,20,21]. Additionally, MNs can be efficiently loaded drugs with aromatic structures through π-π stacking, and can simultaneously deliver drugs to the tumor site [22]. Our previous study reported a melanin nanoprobe targeting PSMA performed successfully for PET/MR/PA imaging and combined therapy in PSMA-positive mouse model, which displayed enhanced permeability and retention (EPR) effects and targeting ability, long-acting properties at the tumor region [16,23]. Therefore, we assumed that combining FAPI with melanin nanoparticles and modifying them with radioactive isotopes and various metal elements could achieve the construction of highly specific targeted FAPI multimodal imaging and treatment integrated nanoprobes, including PAI, MRI, PET/CT, TRT and PTT, which may promote the application of nanoprobes in medicine.

**Scheme 1:**
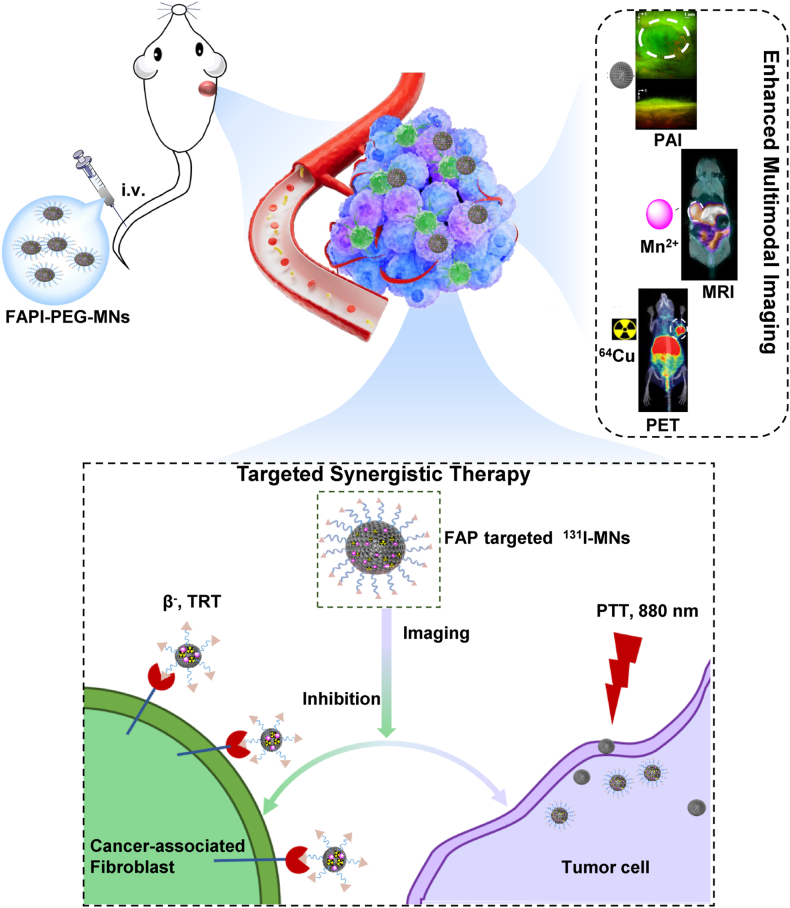
Schematic illustration of the theranostic nanoplatform FAPI-PEG-MNs for PET/MR/PA multimodal imaging and radioisotope-photothermal synergistic therapy of glioblastoma.

Herein, we prepared a novel type of ultrasmall melanin nanoparticles from bio-organic melanin analogues by ultrasonic crushing method and modified with polyethylene glycol (PEG-MNs). To endow melanin nanoparticles with targeting functionality, FAPI was coupled on the surface of MNs to form versatile nanoprobe (FAPI-PEG-MNs). Subsequently, the nanoprobe efficiently chelated positron nuclide ^64^Cu and efficiently chelated the paramagnetic metal Mn^2+^are used for enhanced the imaging effect of PAI, PET/CT, MRI, respectively. Most importantly, due to high photothermal efficiency of MNs upon 808 nm laser irradiation, we simultaneously utilized beta-emitting radionuclides ^131^I to label nanoprobe for the combine therapy of TRT and PTT. Based on the excellent stability targeting of nanoprobe, we investigate the targeting, multimodal imaging effects, and synergistic effects of the combined treatments (Scheme 1). Overall, our study demonstrated that the versatile nanoplatform could realize the targeting, PET/MR/PA multimodal-imaging, and TRT-PTT synergistic therapy, which exhibited great potential as specific theranostic nanoplatform of glioblastoma.

## 2 Results and discussion

### 2.1 Synthesis and characterization of FAPI-PEG-MNs nanoparticles

**Fig. 1:**
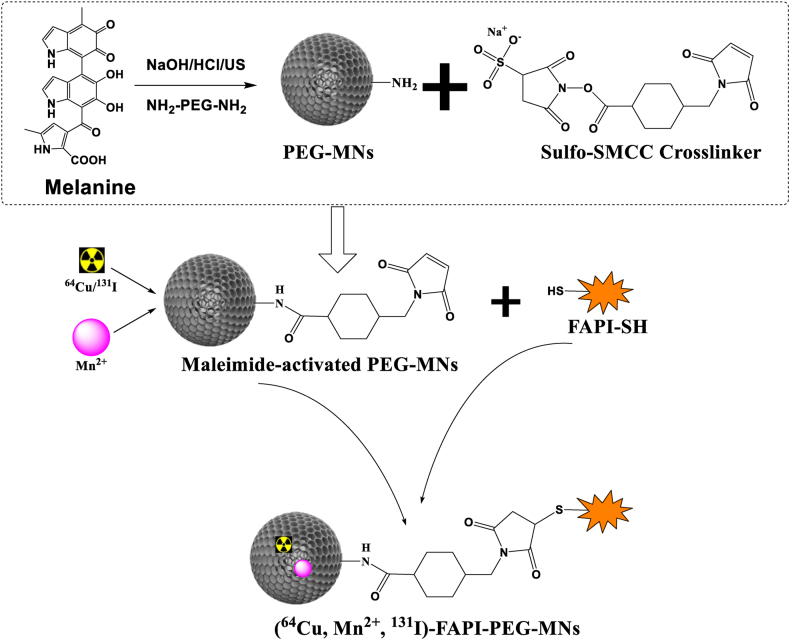
Schematic illustration of the FAPI-PEG-MNs nanoparticles synthesis process.

**Fig. 2:**
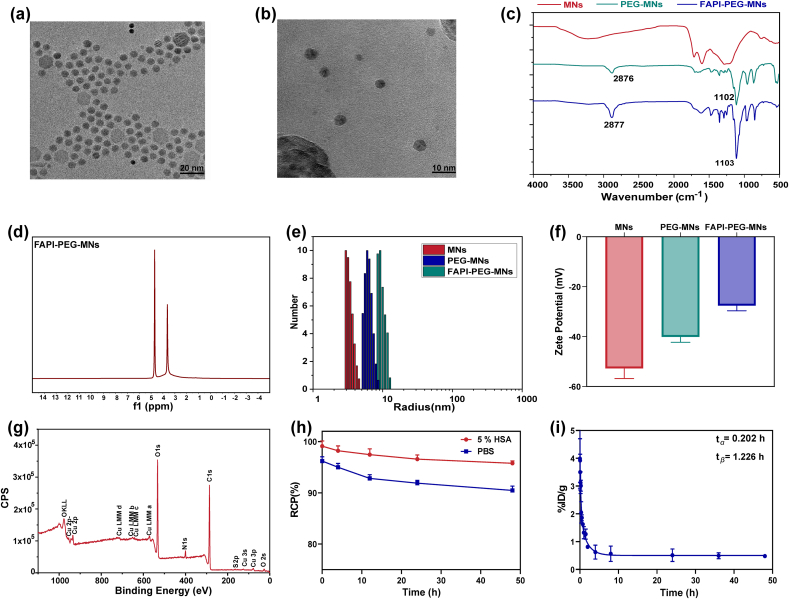
Characterization of FAPI-PEG-MNs nanoparticles. TEM image of a) MNs (scale bar = 20 nm) and b) FAPI-PEG-MNs (scale bar = 10 nm). (c) FT-IR spectra of MNs, PEG-MNs and FAPI-PEG-MNs aqueous dispersions. d) ^1^H NMR spectra of PEG-MNPs in D_2_O. (e) The hydrodynamic size of MNs, PEG-MNs and FAPI-PEG-MNs nanoparticles measured by DLS. f) Zeta potentials of MNs, PEG-MNs and FAPI-PEG-MNs nanoparticles in aqueous solution. g) The XPS of Cu-FAPI-PEG-MNs. h) The in vitro stability of ^64^Cu labeled Mn-FAPI-PEG-MNs in PBS (pH = 7.4) and 5 % HSA. i) The pharmacokinetic study of (^64^Cu, Mn)-FAPI-PEG-MNs in KM mice.

The synthesis procedure of FAPI-SH is shown in Fig. S1. The high-performance liquid chromatography (HPLC) and electrospray ionization mass spectrometry (ESI-MS) results showed that the peak time of FAPI-SH was 6.335 min, its purity was greater than 95 %, and a clear product peak at 575 (Fig. S2), which proved that FAPI-SH was successfully synthesized and its chemical purity met the requirements. The ultrasmall and water-soluble MNs were prepared according to a previously reported synthesis method (Fig. 1) [16]. Transmission electron microscopy (TEM) observation revealed MNs nanoparticles have round-like and regular morphology and good dispersibility in aqueous solution with approximate size of ∼20 nm (Fig. 2a). The surface polarity of MNs changes after binding of metal ions, the diamino polyethylene glycol (PEG) reacts with the dihydroxyindole/indoquinone group of MNs greatly increase the dispersibility and stability of the nanoparticles in solution [23]. As depicted in Fig. 1, FAPI-PEG-MNs were composed of FAPI-SH conjugated to the surface of amino-PEG melanin by amino-sulfhydryl cross-linking agent (Sulfo-SMCC) coupling chemistry (Fig. S3). The morphology and structure of FAPI-PEG-MNs did not change significantly after modification, which would favor tumor accumulation through EPR effect and positive targeting [24] (Fig. 2b). As shown in fourier transform infrared (FT-IR) spectra (Fig. 2c), the FT-IR spectra of MNs, PEG-MNs, and FAPI-PEG-MNs were similar to each other, and the characteristic absorption peaks at 2876 cm^−1^ and 1102 cm^−1^, which can be ascribed to the stretching vibration of alkyl C–H and C–O–C of the PEG molecules, respectively. Meanwhile, the ^1^H nuclear magnetic resonance (^1^H NMR) spectrum of PEG-MNs with D_2_O as dispersion solution shows an emerging peak at 3.5 ppm, which represented the -OCH_2_CH_2_O- group in the PEG molecule (Fig. 2d–S4a, and 4b).

After PEG modification, the hydrodynamic diameter of MNs were 4.05 ± 0.5 nm, which slightly increased to 6.3 ± 1.06 nm after PEG modification, while the hydrodynamic diameter of FAPI-PEG-MNs increased to 10.47 ± 1.44 nm according to dynamic light scattering (DLS) measurement (Fig. 2e), but remained within the appropriate diameter of nanoparticle [25], which would be favorable for tumor accumulation via EPR effect [24]. With the modifications of PEG and FAPI, the surface potential of FAPI-PEG-MNs increased to −27.50 ± 2.15 from −52.69 ± 4.12 of MNs (Fig. 2f), probably owing to the charge shielding effect caused by FAPI-SH, which was beneficial to nanoprobe for their high dispersity and stability in vivo [26]. The X-ray photoelectron spectroscopy (XPS) was used verify to showed the elemental distribution of Cu-FAPI-PEG-MNs (Fig. 2g), indicating that the nanoprobe contains a large amount of Cu^2+^ ions excepting the relevant elements of FAPI-PEG-MNs, and providing a basis for the labeling of the nanoprobe. Moreover, The XPS results for the elemental distribution of iodine ions and MNs have been demonstrated previous literature [16,27].

In this study, (^64^Cu, Mn)-FAPI-PEG-MNs was successfully synthesized with the labeling rate reached 92 % as detected by radio-thin-layer chromatography (TLC) (Fig. S5a). As shown in Fig. 2h, the stability of (^64^Cu, Mn)-FAPI-PEG-MNs were slightly decrease after incubated with 0.01 M PBS solution (pH = 7.4) and 5 % HSA solution for 4 h at room temperature, but its stability was still greater than 90 % after 48 h of incubation. The labeling rate of ^131^I in the nanoprobe was higher than 99 % (Fig. S5b). The elimination of radioactivity from the blood as the a function of time following i.v. (tail vein) injection of (^64^Cu, Mn)-FAPI-PEG-MNs is shown in Fig. 2i. The data were fitted to a two-compartment pharmacokinetic model since (^64^Cu, Mn)-FAPI-PEG-MNs demonstrated a biphasic elimination profile characterized by a rapid distribution phase (α-phase) and a slower elimination (β-phase). As shown in Fig. 2i and Eq. (1), the half-life of α-phase was 0.202 h, and the half-life of β-phase was 1.226 h in (^64^Cu, Mn)-FAPI-PEG-MNs, indicating the nanoprobe could be quickly metabolized from blood.

(1) Ct = 0.503 + 23.78∗e^−3.42t^ + 13.46∗e^−0.57t^

### 2.2 Targeting ability and cytotoxicity of FAPI-PEG-MNs in vitro

**Fig. 3:**
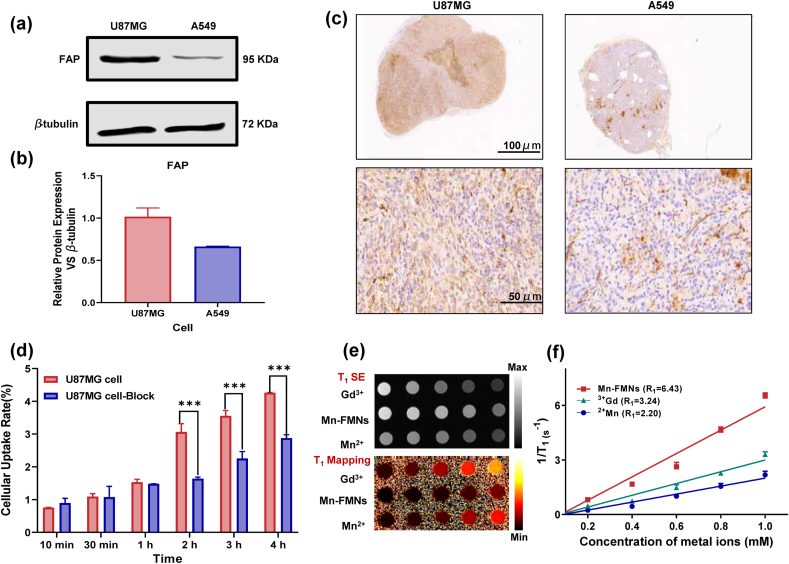
Targeting ability and MRI performance in vitro. a) The expression level of FAP in U87 MG and A549 cancer cells. b) Representative FAP expression was presented as ratios of FAP to β-tubulin expression by western blotting analysis. All data were represented as mean ± SD (n = 3). c) Immunohistochemical staining of U87 MG and A549 tumor, Scale bars = 1000 μm, 50 μm. d) The radioactive uptake of (^64^Cu, Mn)-FAPI-PEG-MNs in U87 MG cells at different time points. e) In vitro T_1_-weighted MRI images of Gd-DTPA, MnCl_2_ and Mn-FAPI-PEG-MNs with various concentrations (2.0, 1.0, 0.25, 0.125, and 0.0625 mg mL^−1^) at 1.5T scanner. f) The linear relationship of T_1_ relaxation rates (1/T_1_, s^−1^) was measured based on various concentrations of Gd^3+^, Mn^2+^ and Mn-FAPI-PEG-MNs in vitro.

To assess the ability of (^64^Cu, Mn)-FAPI-PEG-MNs targeting FAP, we evaluated FAP expression in U87MG and A549 cells using western blot (WB) and immunohistochemistry (IHC) staining, and subsequently conducted cellular uptake experiments. As shown in Fig. 3a, WB analysis revealed significant FAP expression in U87MG cells and low expression in A549 cell. The relative expression of FAP to β-tubulin was 1.02 ± 0.10 in U87MG cells and 0.66 ± 0.01 in A549 cells, indicating a higher level of FAP expression in U87MG cells (Fig. 3b). Furthermore, IHC staining results that a large brown distribution in the U87 MG tumor slices, compared with only a small amount of brown distribution in the A549 slices. The results further confirmed the high level of FAP expression in U87 MG tumor xenografts, consistent with the results of WB results (Fig. 3c). Meanwhile, it was also found to have higher expression on the liver, probably due to the metabolism of the nanoprobe in the mice via the hepatobiliary system, and the probe accumulated on the liver (Figs. S6a and S6b). Additionally, higher FAP expression was observed in the liver, which metabolism of the nanoprobe in the hepatobiliary system (Figs. S6a and S6b).

For cellular uptake experiment. (^64^Cu, Mn)-FAPI-PEG-MNs showed significantly higher uptake in FAP-positive U87 MG cells than in FAP-negative A549 cells, and the difference was statistically significant (Fig. S7). The uptake of (^64^Cu, Mn)-FAPI-PEG-MNs in U87 MG cells increased from 10 min to 4 h (Fig. 3d). The highest uptake of (^64^Cu, Mn)-FAPI-PEG-MNs was observed at 4 h (4.26 ± 0.40 %), which could be blocked with excess non-radiolabeled precursor (2.87 ± 0.07 %), indicating (^64^Cu, Mn)-FAPI-PEG-MNs has a specific targeting ability for FAP-overexpressing tumor cells. Furthermore, cell cytotoxicity assays showed that the cell viabilities of both A549 and Hela cells still remained above 95 % after incubation for 24 h, indicating FAPI-PEG-MNs have minimal cytotoxicity in cell (Figs. S8a and S8b).

### 2.3 In vivo assessment of PET/CT imaging

Nuclear medicine imaging technology enables precise positioning and quantification of targeted regions, providing critical information for personalized diagnosis and treatment. The approach plays a pivotal role in improving patient survival rates. As demonstrated in Figs. S10a and S10b, the PET/CT images of the U87 MG mice revealed clear tumor accumulation over time (Figs. S9a and S9b). The radioactivity of (^64^Cu, Mn)-FAPI-PEG-MNs predominantly concentrated in the heart, liver, spleen, intestine and tumor, with metabolism occurring mainly via the hepatobiliary system—consistent with other melanin-based nanomaterials [28]. The uptake of nanoprobes at the tumor site progressively increased with time, and eventually reached 4.70 ± 0.05 %ID/g at 24 h which was mainly attributed to the combination of the active targeting and the EPR effect of nanoparticles. The tumor-to-muscle (T/M) and tumor-to-liver (T/L) ratios were 15.42 ± 1.06 and 0.52 ± 0.02 at 24 h, respectively (Fig. S12). In A549 tumor-bearing mice with FAP-negative, no significant uptake of (^64^Cu, Mn)-FAPI-PEG-MNs was observed in the tumor region (Fig. S10).

**Fig. 4:**
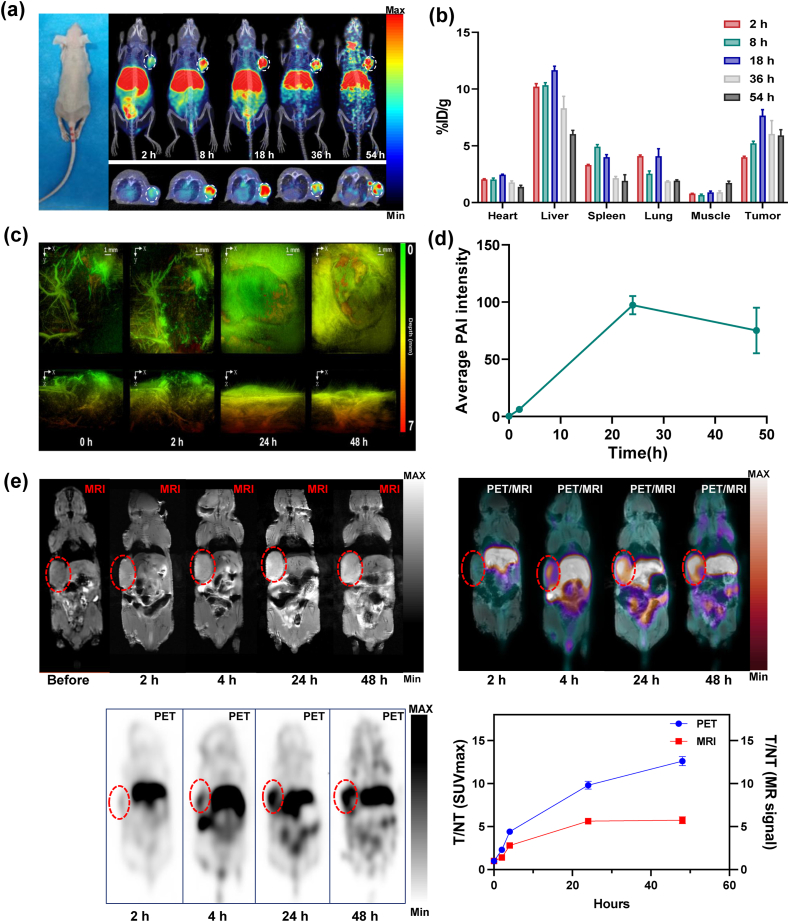
In vivo multimodality imaging. a) Micro-PET/CT images of U87 MG tumor-bearing mice (white dashed circles) at different times after intravenous injection of (^64^Cu, Mn)-FAPI-PEG-MNs. b) The ROI analysis of (^64^Cu, Mn)-FAPI-PEG-MNs in the major organs of U87MG tumor model. c) PA images of the U87 MG tumor (white dashed circles) before and after intravenous injection of FAPI-PEG-MNs. d) Quantitative analysis of enhanced PAI signal of U87 MG tumor before and after intravenous injection of FAPI-PEG-MNs. e) PET/MRI images and the T/NT ratios of SUVmax of U87 MG tumor (red dashed circles) before and after intravenous injection of (^64^Cu, Mn)-FAPI-PEG-MNs.

According to research reported that the abundant extracellular matrix (ECM) in tumors can hinder effective probe retention and penetration while exacerbating the hypoxic tumor microenvironment. These factors collectively reduce the imaging and therapeutic efficacy of many types of probes [29,30]. Hyaluronic acid and collagen are major components of the extracellular matrix in tumors, and their overexpression in tumor tissues leads to elevated interstitial fluid pressure and hinders nanoparticle penetration into solid tumors [30,31]. Collagenase and hyaluronidase can effectively degrade collagen and hyaluronic acid, which leads to relaxation of extracellular matrix structure and promotes deep penetration of nanoparticles into solid tumors [30,32]. In the U87 MG model, pretreatment with collagenase and hyaluronidase did not significantly alter the radiological distribution or metabolism of (^64^Cu, Mn)-FAPI-PEG-MNs compared to untreated models (Fig. 4a and S11a). However, the uptake of (^64^Cu, Mn)-FAPI-PEG-MNs in tumor was significantly increased, reaching 7.65 ± 0.25 %ID/g and 5.2 ± 0.03 %ID/g, respectively (Fig. 4b and S11b). Meanwhile, the (^64^Cu, Mn)-FAPI-PEG-MNs was gradually metabolized in the liver, with a notable enhancement in the tumor-to-non-tumor (T/NT) ratio (Fig. 4b–S12a, and S12b). All these results aff irm that (^64^Cu, Mn)-FAPI-PEG-MNs is highly specific to U87 MG malignant tissues. The prolonged retention of (^64^Cu, Mn)-FAPI-PEG-MNs in tumor region facilitates flexible adjustment of the imaging time, which can improve radionuclide targeting imaging and enable long-term imaging monitoring of tumor treatment.

### 2.4 In vivo assessment of PAI

Based on the inherently excellent PAI performance of the FAPI-PEG-MNs, we performed the PAI study of tumor in vivo [33]. 200 μL of FAPI-PEG-MNs was injected into each mouse by the tail vein, and the PA signals were acquired at different time intervals (0 h (before injection), 2 h, 24 h, and 48 h). As shown in Fig. 4c, the PA signal at the tumor site was weak prior to the injection of FAPI-PEG-MNs. The signal intensity in the tumor region of U87 MG mice increased, likely due to the accumulation of FAPI-PEG-MNs at the tumor site via EPR effect and active targeting FAP. Moreover, the maximum photoacoustic intensity in tumor was detected at 24 h post-injection, and followed by a gradual decline from 24 to 72 h, suggesting a prolonged retention of FAPI-PEG-MNs in the tumor (Fig. 4c and d). These findings demonstrate that FAPI-PEG-MNs possess excellent PAI capabilities in FAP-overexpressing tumors and could be potentially serve as an effective PAI agent for tumor theranostics.

### 2.5 In vitro and in vivo assessment of PET/MRI

MRI can clearly show the tumor structure and boundary with high spatial resolution and can be used to guide surgery. The metal manganese has been used to shorten the T_1_ longitudinal relaxation time of water protons. Mn^2+^ ions as a natural cellular component with are highly paramagnetic, have good biosafety and avoid long-term side effects [34]. At beginning of the multimodal imaging research, we detected the T1-weighted MRI images of different concentrations of Mn-FAPI-PEG-MNs in vitro through comparative studies (Fig. 3e). Meanwhile, the same concentration gradient of manganese chloride and a commercial MRI contrast agent of Gd-DTPA were selected as controls. As the concentration of Mn-FAPI-PEG-MNs increased, the MR signals showed significant enhancement, and was higher than that of MnCl_2_ and the Gd-DTPA solution at the same concentration, which attribute to the strong chelation among Mn^2+^ ions and the plentiful catechol contained of MNs [35]. The R_1_ relativity of the Mn-FAPI-PEG-MNs was extracted from the measured T_1_ data and plotted in Fig. 3f. The measured R_1_ relativities were 2.20, 3.24, and 6.43 mM^−1^s^−1^ for the MnCl_2_, Gd-DTPA, and Mn-FAPI-PEG-MNs, respectively. The highest R1 value of Mn-FAPI-PEG-MNs was that of Gd-DTPA, indicating that the nanoparticles generate a high magnetic field gradient on their surface.

For PET/MRI in vivo, (^64^Cu, Mn)-FAPI-PEG-MNs were injected into the U87 MG tumor-bearing mice and the tumor was weighted by coronal T_1_ images at appropriate time intervals before and after injection (2 h, 4 h, 24 h and 48 h) to acquire magnetic resonance images the T/NT ratios of the maximum standardized uptake value (SUVmax). As the shown in Fig. 4e, the MR signal at the tumor site was substantially increased over time following the injection of (⁶⁴Cu, Mn)-FAPI-PEG-MNs, exhibiting significantly higher intensity compared to pre-injection levels. Subsequently, the MR signal of the (^64^Cu, Mn)-FAPI-PEG-MNs in tumor tissue peaked at 24 h and slightly decreased by 48 h, a trend consistent with the observations in PAI. Statistical analysis of the SUVmax and MR signals at the tumor site revealed that the T/NT ratio for both signals increased over time, while the MR signal intensity in the tumor region declined after 24 h. Additionally, the MR signal in the liver gradually increased, indicating prolonged nanoparticle circulation and reduced rapid clearance. These findings suggest that (⁶⁴Cu, Mn)-FAPI-PEG-MNs are primarily excreted through the biliary metabolic pathway, minimizing the potential side effects associated with nanoparticle accumulation in vivo.

### 2.6 Combined radionuclide therapy and phototherapy synergistic effect of FAPI-PEG-MNs in vivo

**Fig. 5:**
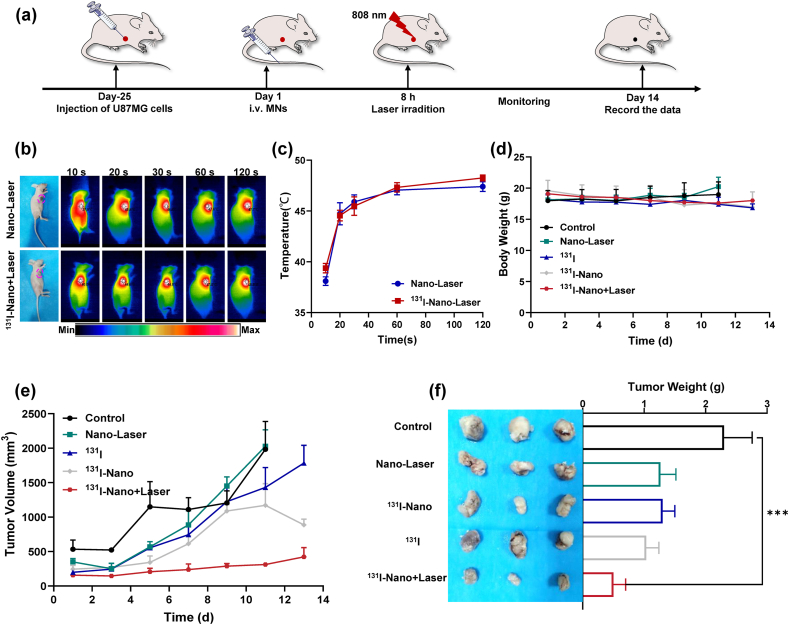
*In vivo* assessment of radioisotope-photothermal synergistic therapeutic effect. a) The schematic illustration of the tumor therapeutic profile. b) IR thermal images of tumor-bearing mice exposed to the NIR laser after injection with FAPI-PEG-MNs and ^131^I-FAPI-PEG-MNs. c) The temperature changes of tumor-bearing mice exposed to the NIR laser after injection with FAPI-PEG-MNs and ^131^I-FAPI-PEG-MNs. d) Body weight curves of U87 MG tumor-bearing mice under different treatments over 13 days. d) Tumor growth curves of different treatments. f) Photographs of tumors removed from mice in different groups after 13 days and tumor weight of different treatments.

Encouraged by the high photothermal conversion efficiency and satisfying tumor uptake ability [16]. FAPI-PEG-MNs can potentially be used for tumor therapies through the combination of PTT and TRT. We proceeded to assess in vivo effects of FAPI-PEG-MNs in the synergistic TRT/PTT using the U87 MG tumor-bearing mouse model (Fig. 5a). Briefly, the mice were randomly divided into five groups, including Control, Nano-Laser, free ^131^I, ^131^I-Nano, and ^131^I-Nano + Laser. After the injection for about 8 h, irradiated with 808 nm laser to the tumor sites for Nano-Laser and ^131^I-Nano + Laser, and the full-body infrared thermal images and the tumor temperature were real-time monitor by a thermal imaging camera (Fig. 5b–c). Notably, the surface temperature of tumors sites after irradiation increased from ∼37 °C to ∼47 °C at 120 s, and maintained at ∼48.0 °C during laser irradiation corresponding to the brighter red color in thermal images. These results favored the potential application of FAPI-PEG-MNs in photothermal therapy. For the next 13 days, tumor growth was monitored every 2 days. As show in Fig. 5e, the tumor volume gradually increased as time goes by of only injected with PBS solution, Nano-Laser, and free ^131^I group, indicating that laser irradiation or free radionuclide alone is ineffective for tumor treatment. In contrast, the tumor growth in the ^131^I-Nano + Laser group was inhibited within 13 days, and was approximately 21.3 % of the control group (422 mm^3^ relative to 1982 mm^3^, Fig. 5e). Furthermore, the body weight remained stable across all 5 groups during the treatment period (Fig. 5d). The tumor suppression outcomes of FAPI-PEG-MNs in all groups are summarized in Fig. 5f. While tumor growth varied among the groups, the results clearly demonstrate that FAPI-PEG-MNs possess effective tumor suppression abilities in vivo in vivo through synergistic PTT/TRT.

**Fig. 6:**
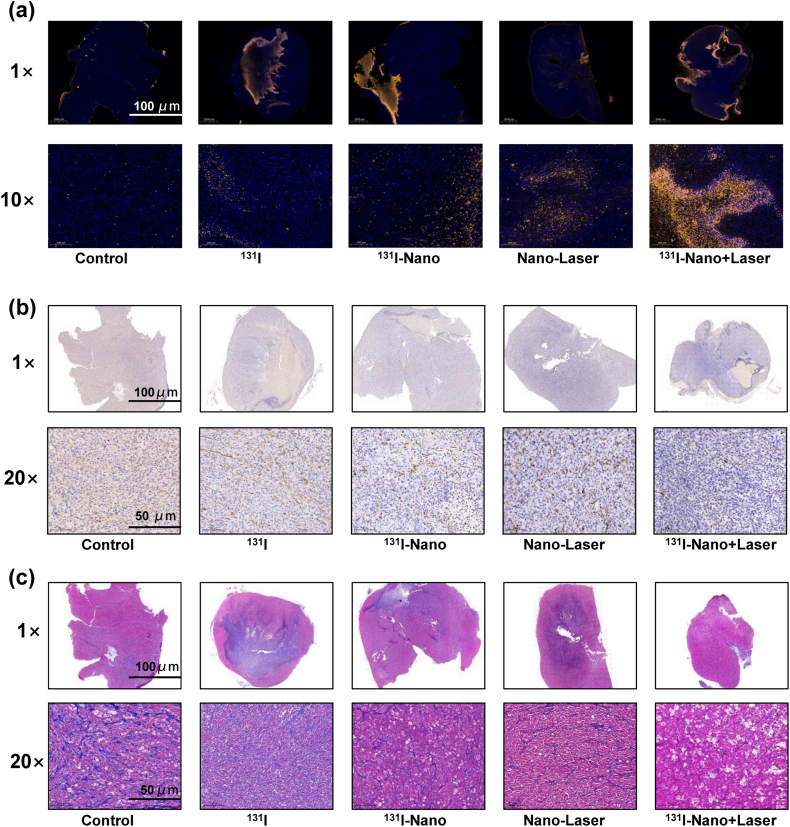
Histological analysis of the tumor slices with different treatments via a) TUNEL, b) α-SMA and c) Masson staining. Scale bars = 1000 μm (1 × ), Scale bars = 100 μm (10 × ), Scale bars = 50 μm (20 × ).

To further elucidate the histological changes in tumors of mice receiving different treatments, tumor samples were collected and analyzed. Tumor sections were subjected to terminal-deoxynucleotidyl transferase-mediated nick end labeling (TUNEL), Masson, and α-smoothened muscle actin (α-SMA) staining (Fig. 6). The TUNEL assay showed most cancer cells apoptosis in the group 5, with only a few apoptotic cells in the tumors from the group 1. Varying degrees of apoptotic cells were found in the group 2, group 3, and group 4 (Fig. 6a). Immunohistochemical staining for α-SMA revealed a marked reduction in the intensity and number of positively stained cells on group 5 of ^131^I-Nano + Laser after treatment of day 13, compared to the saline control group (group 1) (Fig. 6b). In addition, ewer collagen fibers and significantly increased apoptosis were observed in the same therapeutic group, consistent with the TUNEL results. These findings indicate that FAPI-PEG-MNs can effectively induce tumor cell damage under radionuclide therapy combined with 808 nm laser irradiation (Fig. 6c). Together, these results highlight the superior therapeutic efficacy of dual therapy with FAPI-PEG-MNs. Thus, the combination of photothermal and radioisotope therapies demonstrates enhanced effectiveness in cancer treatment.

## 3 Conclusion

In summary, a theranostic nanoplatform based on melanin nanoparticles modified with FAP-targeting small molecules was successfully developed for PA/MR/PET multimodal imaging and radionuclide-photothermal synergistic therapy of solid tumor. The nanoplatform exhibited excellent biocompatibility, stability, and specificity in vitro. With strong NIR absorption and metal chelating ability, the nanoplatform applied to PAI/MRI/PET imaging. When the nanoplatform was injected into tumor-bearing mice, it was confirming the nanoplatform can efficiently targeting accumulation and exhibited prolong retention at the tumor site. Simultaneously, the combination of TRT and PTT using the nanoplatform significantly inhibited the tumor growth of U87 MG tumor model, achieving effective synergistic therapy with minimal side effects in comparison to individual therapy. Our results offer a way to harness melanin nanoparticles-based theranostic agents to achieve enhanced multimodal imaging and TRT-PTT synergistic therapy of solid tumor.

## 4 Experimental

**Materials and Reagents**: Melanin (No. 8049-97-6), hydrochloric acid (No. 7647-01-0), 4-(N-maleimidomethyl) cyclohexane-1-carboxylic acid sulfosuccinimidyl ester sodium salt (Sulfo-SMCC, No. 92921-24-9), sodium acetate (NaOAc, No.6131-90-4), N-bromosuccinimide (NBS, No. 128-08-5) purchased from Sigma-Aldrich (St Louis, MO, USA). Amine-PEG-NH_2_ (mPEG_5000_-NH_2_, 5 kDa, No. 24991-53-5), manganese chloride (MnCl_2_, No. 7773-01-5) was obtained from Beijing InnoChem Science & Technology Co., Ltd (Beijing, China). Sodium hydroxide (NaOH, No. 1310-73-2), sodium chloride (NaCl, CAS No. 7647-14-5), potassium chloride (KCl, No. 7447-40-7), potassium dihydrogen phosphate (KH_2_PO_4_, No. 7778-77-0), disodium phosphate (Na_2_HPO_4_, No. 7558-79-), sodium chloride (Na_2_HPO_4_), and ethylenediaminetetraacetic acid disodium salt (EDTA-2Na, No.6381-92-6) were purchased from Sinopharm Chemical Reagent Co (Shanghai, China). Sodium dodecyl sulfate–polyacrylamide gel electrophoresis (SDS-PAGE) equipment was purchased from Kangweishiji Biotechnology Co., Ltd (Beijing, China). The anti-FAP monoclonal antibody (No. ab207178) was purchased from Abcam (Cambridge, UK). Positron nuclide ^64^Cu was produced by Peking University Cancer Hospital nuclear medicine department cyclotron team, using the HM-20 cyclotron, through the reaction of ^64^Ni (p,n) ^64^Cu (specific activity approximately 5.6 GBq/μmol). High-purity therapeutic nuclide ^131^I was purchased from Atomic High-Tech Co., Ltd. (Beijing, China). All chemicals and reagents were high purity chemicals and used as received without further purification.

**Fabrication and characterization of FAPI-PEG-MNs**: **i) Synthesis and Modification of MNs:** The ultrafine melanin nanoparticles (MNs) were prepared by ultrasonic fragmentation in accordance to previous studies [16]. 15 mg of melanin was completely dissolved in 3 mL of a 0.2 M sodium hydroxide (NaOH) solution under vigorous stirring. Subsequently, 1.5 mL of a 0.2 M hydrochloric acid (HCl) solution was added within 1 min using ultrasonic pulverizer at 160 W power. After cooling, 0.95 mL HCl solution was added to neutralize the pH. The resulting bright black solution was centrifuged (Amicon centrifugal filter MWCO = 30 kDa) and washed multiple times to remove free sodium ions (Na^+^), chloride ions (Cl^−^), and unpolymerized melanin molecules. The MNs dispersion was adjusted to pH 9 with 0.1 M NaOH and added dropwise to a solution containing 10 mg of diamino polyethylene glycol (NH_2_–PEG_5000_–MNs). After stirring at room temperature for 12 h, unreactedNH_2_–PEG–MNs was removed by ultrafiltration, yielding NH_2_–PEG–MNs. **ii) Synthesis of FAPI-PEG-MNs:** The modified MNs was dissolved and mixed with amino-sulfhydryl cross-linking agent Sulfo-SMCC (5 μmol), and the reaction was stirred at room temperature for 2 h. Then the aqueous FAPI-SH solution (4 × 10^−3^ M, 0.5 mL) was slowly added dropwise to the above mixture and was carried out overnight at room temperature with stirring. After the reaction was completed, the product was purified using a PD-10 column to remove the excess material and concentrated by ultrafiltration. **iii) Mn**^**2+**^ **chelated FAPI-PEG-MNs:** The method of Mn^2+^ chelated FAPI-PEG-MNs as our previous work [16,23]. Briefly, an excess of aqueous MnCl_2_ (10 mM, 0.5 mL) and the above prepared FAPI-PEG-MNs (4 mg, 1 mL) were mixed and stirred for 2–3 h at room temperature. After the reaction was completed, the unreacted MnCl_2_ was removed by washing three times using an ultrafiltration centrifuge tube (MWCO = 10 kDa) to obtain Mn-FAPI-PEG-MNs. **ⅳ) Characterization of FAPI-PEG-MNs:** the shape and size of nanoparticles were characterized by using high-resolution transmission electron microscopy (TEM, JEM-2100F, 200 kV), X-ray photoelectron spectroscopy (XPS, PerkinElmer PHI-5400), and the samples diluted to 1 mg/mL, prepared using copper mesh membranes. Hydrodynamic diameters were examined by dynamic light scattering (DLS, SLS-5022F, Germany) and Zeta potential measurements were carried out on Zetasizer Nano ZS (Malvern Instruments Ltd., UK); D_2_O was used as the solvent and ^1^H NMR spectra of nanoparticles using nuclear magnetic resonance hydrogen spectrometry (600 MHz, Bruker, USA); Fourier transform infrared (FT-IR) spectroscopic analysis was performed on an infrared spectrophotometer (Nicolet IS 50, Thermo Fisher, USA).

**Radiolabeling and quality control**: **i)**^**64**^**Cu labeling of Mn-FAPI-PEG-MNs:** Radioactive positronium nuclides ^64^Cu (decay half-life: 12.7 h) was produced by Peking University Cancer Hospital using the HM-20 cyclotron by reaction with ^64^Ni (p, n)^64^Cu (specific activity of about 5.6 GBq/μmol). (^64^Cu, Mn)-FAPI-PEG-MNs was labeled and purified as reported previously [19], FAPI-PEG-MNs was incubated with ^64^CuCl solution in 0.1 M sodium acetate (pH 5.5). After 1 h incubation at 40 °C continuous shaking, ethylenediaminetetraacetic acid (EDTA, 4 mM final concentration) was added to the reaction solution to scavenge excess ^64^Cu^2+^ ions before PD-10 purification. The mixture was purified through a PD-10 column with PBS (0.01 M, pH = 7.4) as the elution buffer to obtain (^64^Cu, Mn)-FAPI-PEG-MNs. After purification, a small amount of solution was taken out for analysis of purity with radio-TLC. **ⅱ)** ^**131**^**I labeling of FAPI-PEG-MNs:** High-purity therapeutic nuclide ^131^I was passed through China Atomic HTA Co., Ltd. For the radiolabeling procedures as reported previously [16], ^131^I solution (111 MBq) was diluted with phosphate buffer solution (0.1 M, 500 μL) and mixed with FAPI-PEG-MNs. Following by 20 μL N-bromosuccinimide (NBS, 1 mg/mL) solution and the reaction was immediately timed for 1 min. The purification was quickly collected by using a PD-10 column with PBS buffer. After purification, a small amount of solution was taken out for analysis of purity with radio-TLC. **iii) The stability of (**^**64**^**Cu, Mn)-FAPI-PEG-MNs:** The stability of (^64^Cu, Mn)-FAPI-PEG-MNs (1.85 MBq, 50 μCi) in vivo was measured by incubations in PBS (pH = 7.4) and 5 % HSA solution. Radio-TLC was used to analyze the radiochemical purity of the (^64^Cu, Mn)-FAPI-PEG-MNs at different time points.

### 4.1 Cell experiments

**Cell culture**: Human glioma U87 MG cell line, human lung adenocarcinoma cell line A549 cell line, and human cervical cancer Hela cell line were purchased from the Type Culture Collection of the Chinese Academy of Sciences (Shanghai, China). All cell culture related reagents were purchased from Gibco (Waltham, USA). Cells were cultured in Dulbecco's modified eagle medium (DMEM) containing 10 % fetal bovine serum (FBS) and 1 % penicillin-streptomycin (PS) at 37 °C under a humidified condition of 5 % CO_2_.

**Cytotoxicity assay:** In vitro cytotoxicity of FAPI-PEG-MNs and Mn-FAPI-PEG-MNs was determined in A549 and Hela cells by the MTT assay. A549 and Hela cells were seeded into 96-well plates at 5000 cells per well and incubated with various concentrations of FAPI-PEG-MNs and Mn-FAPI-PEG-MNs (0.125, 0.25, 0.5, 1, and 2 mg/mL) for 24 h. The old medium was removed and washed with fresh medium several times. Finally, 20 μL of MTT solution (5 mg/mL) was added to each well, followed by another 3–4 h of incubation protected from light. The relative viability of the untreated control was normalized to be 100 %, while the media absorbance set as a background control. Each experiment was performed four times.

**Cellular uptake**: U87 MG and A549 cells were plated on 24-well plates (1 × 10^5^ cells per well) 24 h before the experiments. (^64^Cu, Mn)-FAPI-PEG-MNs (74 kBq, 500 μL) in DMEM medium was added into each well, and 20 μg of non-radiolabeled precursor were added to each well for blocking. After incubation at 37 °C for 10 min, 30 min, 1 h, 2 h, 3 h, and 4 h, respectively, the medium was carefully removed and the cells were washed twice with PBS (1 mL × 2). Cell lysis was then carried out using 0.1 M NaOH, and the radioactivity was measured by a γ-counter. 1 % of the radioactivity of (^64^Cu, Mn)-FAPI-PEG-MNs as the reference activity of the assay. This study was repeated four times for each time point.

**Western blot assay in vitro**: In the western blotting assay for quantitative detection of FAP protein expression in cells. U87 MG cells and A549 cells were selected as experimental cells. Total protein concentrations were detected via the BCA protein assay kit. Certain amounts of each protein were separated by sodium dodecyl sulfate-polyacrylamide gel via electrophoresis and transferred onto polyvinylidene difluoride PVDF membrane. The membranes were incubated with primary antibody against FAP(1:1000) at 4 °C overnight, and then followed by the secondary antibody (sheep anti-rabbit IgG) for 2 h. FAP protein levels were detected and scanned on enhanced chemiluminescence instrumentation.

### 4.2 Animal experiments

**Establishment of tumor model**: All animal studies were performed in accordance with the Guidelines for Care and Use of Laboratory Animals of Peking University (Beijing, China) and experiments were approved by the Animal Ethics Committee of the National Regulation of China for the Care and Use of Laboratory Animals and in compliance with the guidelines established by Peking University Cancer Hospital Animal Care and Use Committee (EAEC 2022-01). Female BALB/c nude mice (6–8 weeks) were purchased from Beijing Viton Lever Laboratory Animal Technology Co. Approximately 2 × 10^6^ tumor cells of U87 MG and A549 in 100 μL PBS mixed with Matrigel (BD Biosciences, Franklin Lake, NJ, USA) were subcutaneously injected into the right front flank of the mice. When the tumor had grown 5–10 mm in diameter, the relevant animal experimental studies was performed.

**Pharmacokinetic of (**^**64**^**Cu, Mn)-FAPI-PEG-MNs in vivo**: To investigate the pharmacokinetics profile, healthy female KM mice (n = 4) were intravenously injected with 200 μL of (^64^Cu, Mn)-FAPI-PEG-MNs (3.7 MBq).Blood samples were collected at predefined time points (1 min, 3 min, 5 min, 10 min, 15 min, 30 min, 45 min, 1 h, 2 h, 4 h, 8 h, 24 h, and 48 h) through the orbital vein. The collected blood samples were weighed before prior to radioactivity measurement using a γ-counter. The results were expressed as a percentage of injected dose per gram of organ (%ID/g, mean ± SD) for each time point.

**PET/CT imaging in vivo:** Mice bearing U87 MG and A549 xenograft tumor were intravenously injected with 200 μL of (^64^Cu, Mn)-FAPI-PEG-MNs (7.4 MBq) via the tail vein. Axial and coronal PET/CT images of the tumor were collected each different time point (2 h, 8 h, 18 h, 36 h, and 54 h) after injection, the mice were anesthetized with 1.5 % isoflurane and placed on the imaging bed to perform micro-PET/CT imaging (Super Nova, PINGSENG Healthcare, China). The other two groups of U87 MG mouse were injected with 100 μg of hyaluronidase and collagenase 8 h in advance and then injected with the same dose of (^64^Cu, Mn)-FAPI-PEG-MNs. PET/CT images were performed in static scanning mode for 10 min, and reconstructed with attenuation correction based on the CT data (CT- AC reconstruction). Region of interest (ROI) was depicted on micro-PET/CT data processing software, calculating and analyzing the tumor-to-non-tumor ratio.

**PA performance of FAPI-PEG-MNs in vivo**: The FAPI-PEG-MNs (1 mg. mL^−1^, 200 μL) was intravenously injected into U87 MG tumor-bearing mice (n = 4) via tail vein. Mouse were anesthetized with 1 % isoflurane throughout the experiments, the PA imaging was acquired to cover the subcutaneous tumor regions using 3D real time PA imaging system at various time points (before injection and 2 h, 24 h, and 48 h post-injection). The PA images were acquired with a scanning wavelength of 800 nm, and the scanning time was 10 min. The averaged PA signals of the tumor area were extracted using the system's own image processing software.

**PET/MRI performance of Mn-FAPI-PEG-MNs in vitro and in vivo**: To detect the in vitro T_1_-weighted contrast function of Mn-FAPI-PEG-MNs, Gd-DTPA, and MnCl_2_ were prepared in different concentrations (0.0625, 0.125, 0.25, 1, and2 mg. mL^−1^) and mixed with 1 mL of 1 % agarose gel. T_1_-weighted multilayer scans of the counting tubes were performed using a clinical 1.5T MR scanner with an animal micro-coil. The imaging data were processed using Image J software to calculate R_1_ values and curve fitting results. To evaluate MRI contrast enhancement effect in vivo, the U87 MG tumor-bearing mouse was injected with (^64^Cu, Mn)-FAPI-PEG-MNs (7.4 MBq, 200 μL) via the tail vein, and were anesthetized by intraperitoneal injection of 4 % chloral hydrate. The mice were scanned using a clinical PET/MRI scanner with an animal microcoil in the co-culture unit for T_1_-weighted multilayer scans at various time points (before injection and 2 h, 4 h, 24 h, and 48 h post-injection), with a scanning time of 20 min and an axial position. The images were processed using the software provided with the imaging system. The analysis of the MR signal in the tumor images was conducted with 3D slice software.

**In vivo efficacy and histological staining**: U87 MG tumor-bearing nude mice were used to determine the therapeutic effect of ^131^I-Nano + Laser. The nude mice were randomly divided into five groups (three mice per group): Control (Group 1), Nano-Laser (Group 2), ^131^I (Group 3), ^131^I -Nano (Group 4), and ^131^I -Nano + Laser (Group 5). The first group was injected with 200 μL sterile saline solution via tail vein. The second group was injected with FAPI-PEG-MNs (1 mg. mL^−1^, 200 μL) through tail vein, and the infrared laser (808 nm, 1.3 W/cm^2^) was used to irradiate the tumor site for 20 min at 8 h after injection. The tumor-bearing mice were lightly anesthetized with isoflurane, and the temperature changes of the tumor site were detected by thermography and photographed. The third and fifth groups were injected with ^131^I -FAPI-PEG-MNs (11.1 MBq, 200 μL), and the tumor site of fifth groups was irradiated with infrared laser for 20 min (808 nm, 1.3 W/cm^2^), and the tumor temperature was monitored above 45 °C. The fourth group were injected with ^131^I saline diluent (11.1 MBq, 200 μL). The body weights and tumor volumes were measured and monitored every two days, and the mice were euthanized on day 14 after treatment. Tumor size was calculated by the following formula: volume = short diameter [2] × long diameter/2. To further evaluate the therapeutic effect of the tumors, tumor tissues were fixed with 4 % paraformaldehyde solution for 24 h at room temperature, and then frozen and cut into the slices with a thickness of 6 μm. The sliced tumors were stained with Tunnel, Masson, and α-SMA, followed by observation under a microscope.

**Statistical analysis**: For each experiment, values are displayed as mean ± standard deviation. Statistical analyses were performed using GraphPad Prism 9. Statistical differences were determined using One-way ANOVA for three or more groups. ∗P < 0.05, ∗∗P < 0.01, ∗∗∗P < 0.001, ns = not significant.

## CRediT authorship contribution statement

**Li Wen:** Writing – original draft, Methodology, Investigation, Formal analysis, Data curation. **Chengxue He:** Methodology, Formal analysis, Data curation. **Yanhui Guo:** Writing – review & editing, Validation, Methodology, Formal analysis. **Nina Zhou:** Visualization, Validation. **Xiangxi Meng:** Validation, Software, Formal analysis. **Yuwen Chen:** Software, Methodology, Formal analysis. **Cheng Ma:** Validation, Software, Formal analysis. **Hua Zhu:** Supervision, Project administration, Funding acquisition. **Zhi Yang:** Supervision, Project administration, Funding acquisition. **Lei Xia:** Supervision, Project administration, Conceptualization.

## Declaration of competing interest

The authors declare the following financial interests/personal relationships which may be considered as potential competing interests: No If there are other authors, they declare that they have no known competing financial interests or personal relationships that could have appeared to influence the work reported in this paper.

## Data Availability

No data was used for the research described in the article.
