# Supplementary material for: Strategies for specific multimodal imaging of cancer-associated fibroblasts and applications in theranostics of cancer
Source: Mater Today Bio. 2024 Dec 24;30:101420. doi: 10.1016/j.mtbio.2024.101420 (PMC11745968; doi:10.1016/j.mtbio.2024.101420)
Supplement: Multimedia component 1 [file mmc1.docx]

Supporting Information

# Strategies for Specific Multimodal Imaging of Cancer-Associated Fibroblasts and Applications in Theranostics of Cancer

Li Wen^1,2,^^[[1]](#footnote-1)^*, Chengxue He^1,*^, Yanhui Guo^3,*^, Nina Zhou^1^, Xiangxi Meng^1^, Yuwen Chen^4,5^, Cheng Ma^4,5^, Hua Zhu^1,^^[[2]](#footnote-2)^#, Zhi Yang^1,#^, Lei Xia^1,#^

1. Key Laboratory of Carcinogenesis and Translational Research (Ministry of Education/Beijing), NMPA Key Laboratory for Research and Evaluation of Radiopharmaceuticals (National Medical Products Administration), Department of Nuclear Medicine, Peking University Cancer Hospital & Institute, Beijing 100142, China

2. Department of Nuclear Medicine, Union Hospital, Tongji Medical College, Huazhong University of Science and Technology, Hubei Key Laboratory of Molecular Imaging, Key Laboratory of Biological Targeted Therapy, the Ministry of Education, Wuhan 430022, China

3. Department of Radiology, Peking University Third Hospital, Beijing 100088, China

4. Department of Electronic Engineering, Beijing National Research Center for Information Science and Technology, Tsinghua University, Beijing, 100084, PR China

5. Institute for Intelligent Healthcare, Tsinghua University, Beijing, 100084, PR China


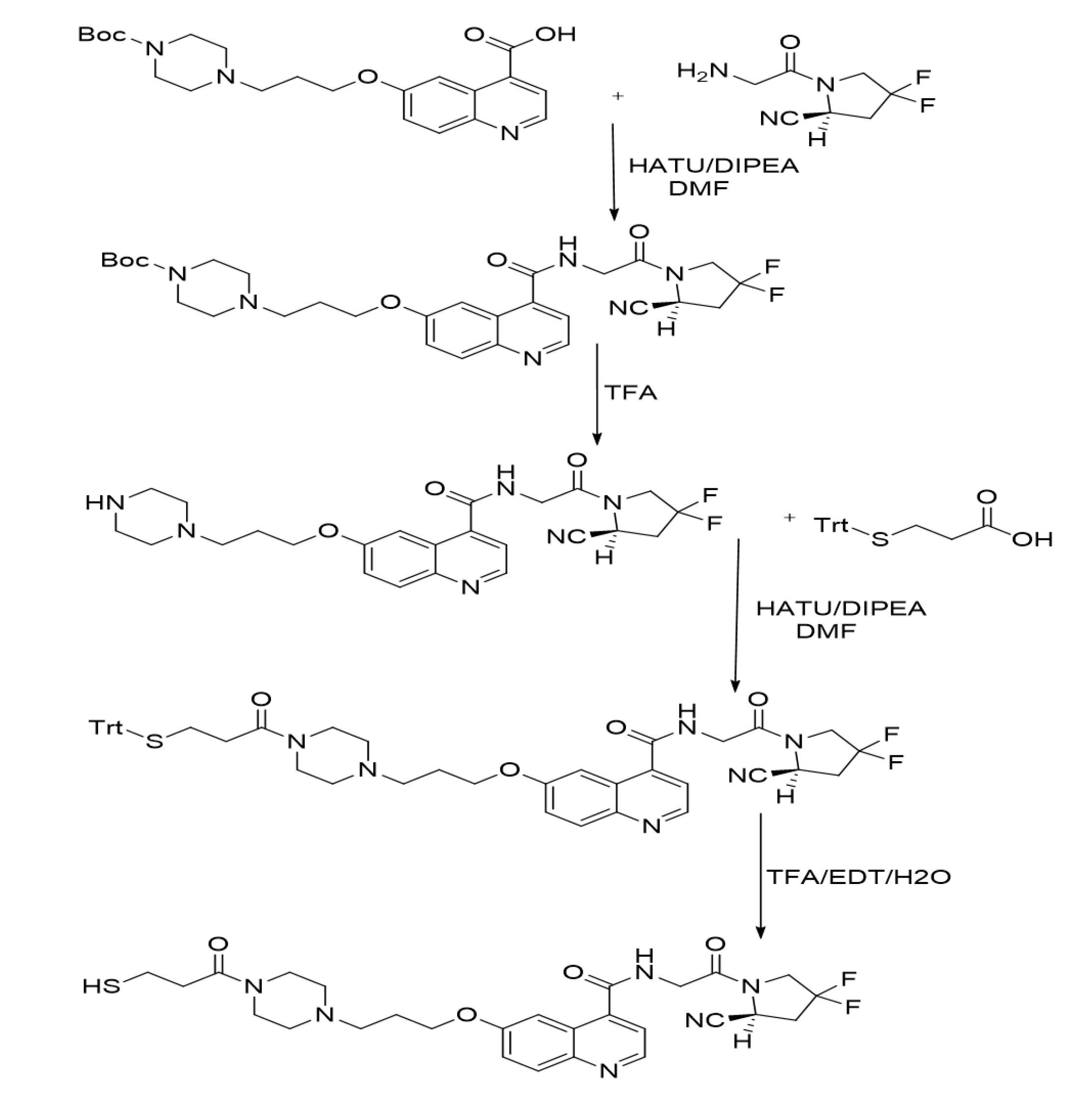


**Figure S1**. Synthetic route of FAPI-SH.


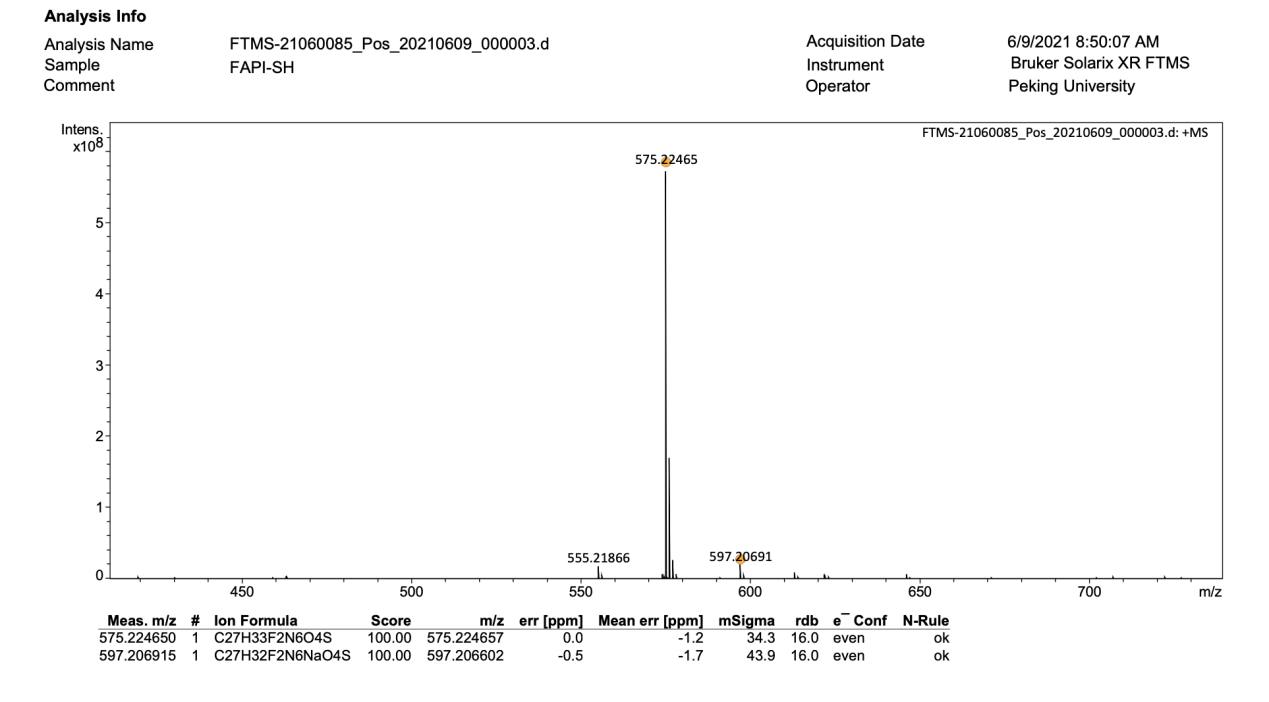


**Figure S2**. The MALDI-TOF-MS of FAPI-SH.


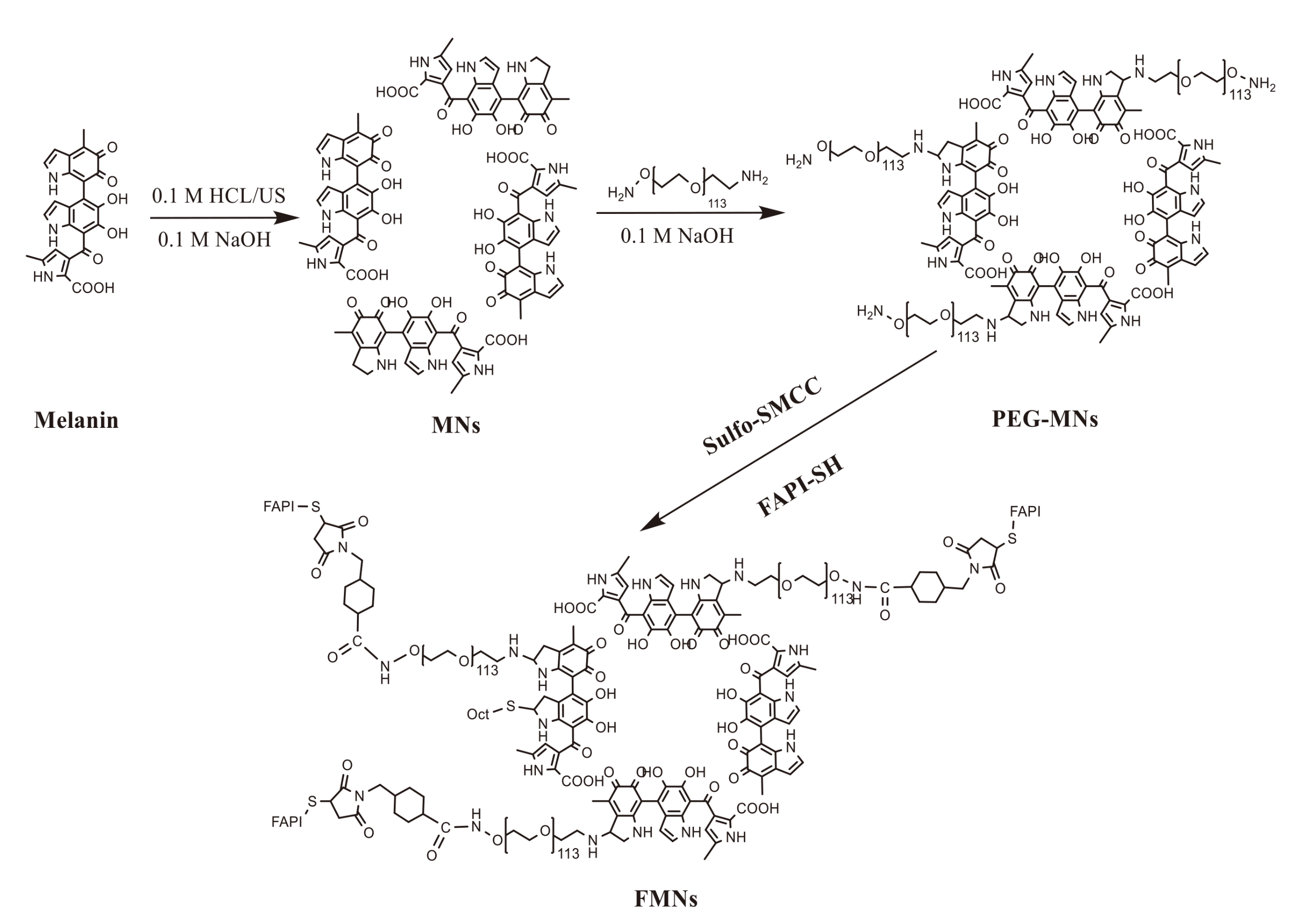


**Figure S3.** Synthetic route of FAPI-PEG-MNs.


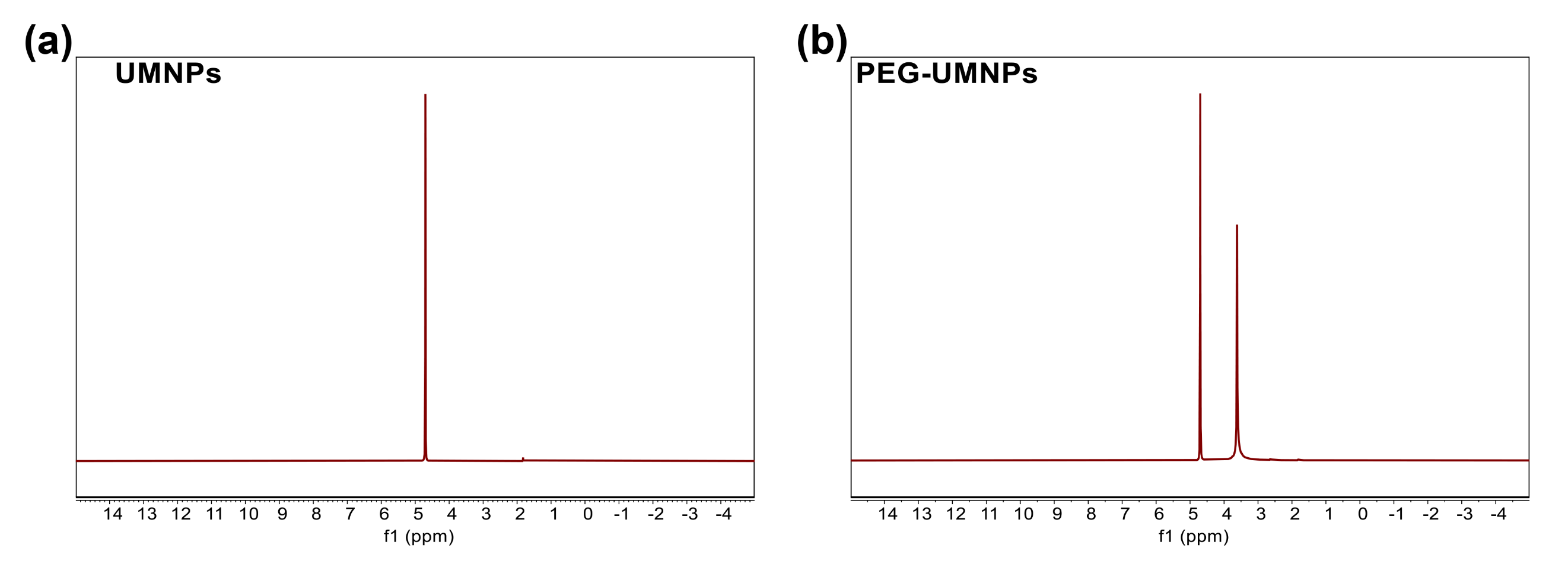


**Figure S4.** ^1^H-NMR spectra of MNs-Ⅱ a) and PEG-MNs b) in D_2_O.


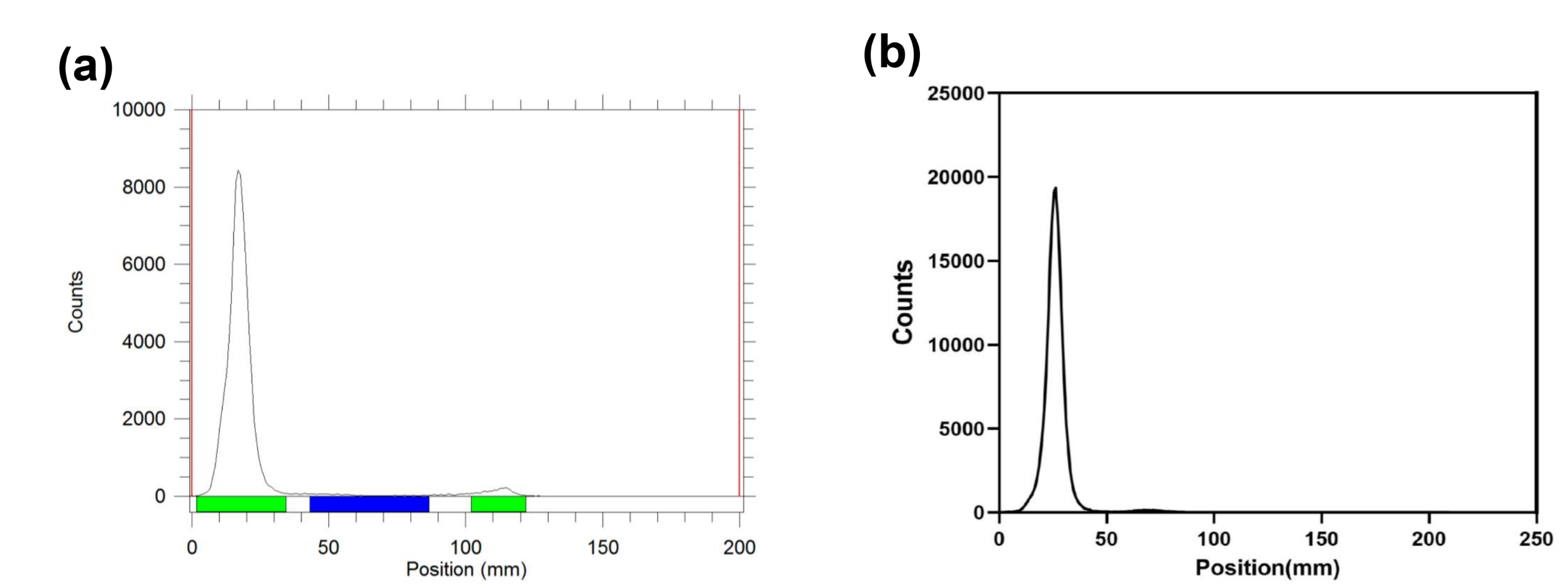


**Figure S5.** Radio-TLC chromatograms of ^64^Cu/^131^I-labeled FAPI-PEG-MNs. a) (^64^Cu, Mn)-FAPI-PEG-MNs before purification; b) The labeling rate of (^131^I, Mn)-FAPI-PEG-MNs.


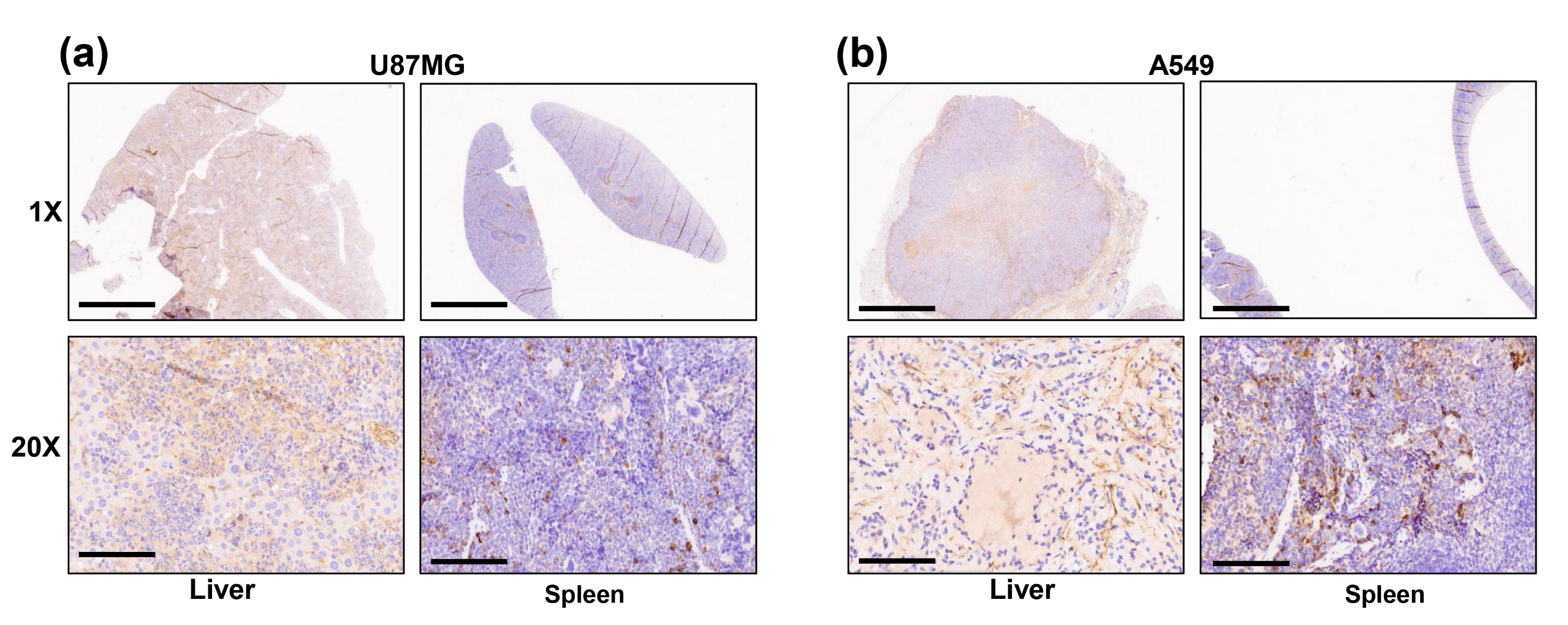


**Figure S6**. The FAPI expression of a) U87 MG and b) A549 tissues of liver and spleen were measured by immunohistochemical staining.


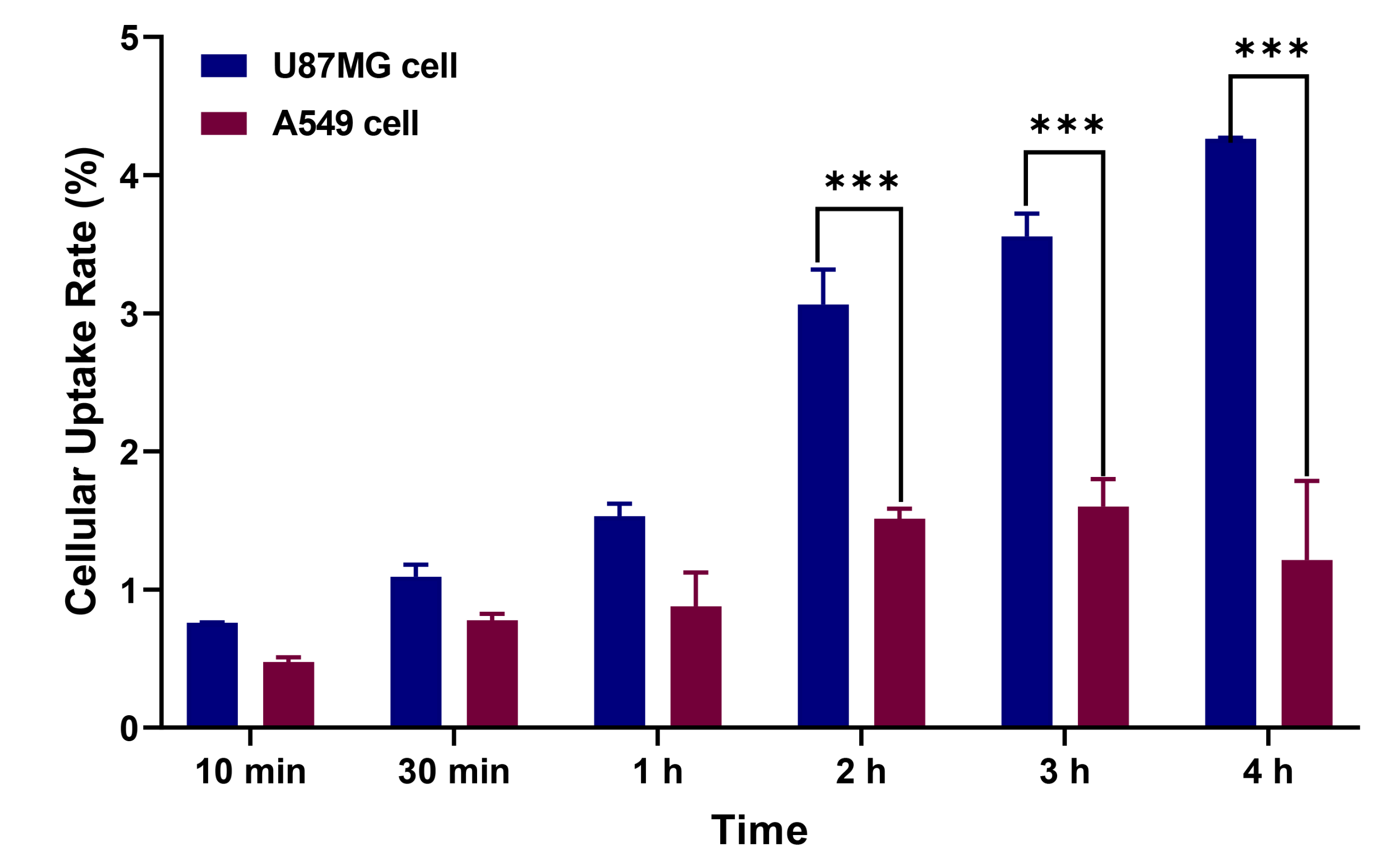


**Figure S7**. The radioactive uptake of (^64^Cu, Mn)-FAPI-PEG-MNs in U87 MG and A549 cells at different time points.


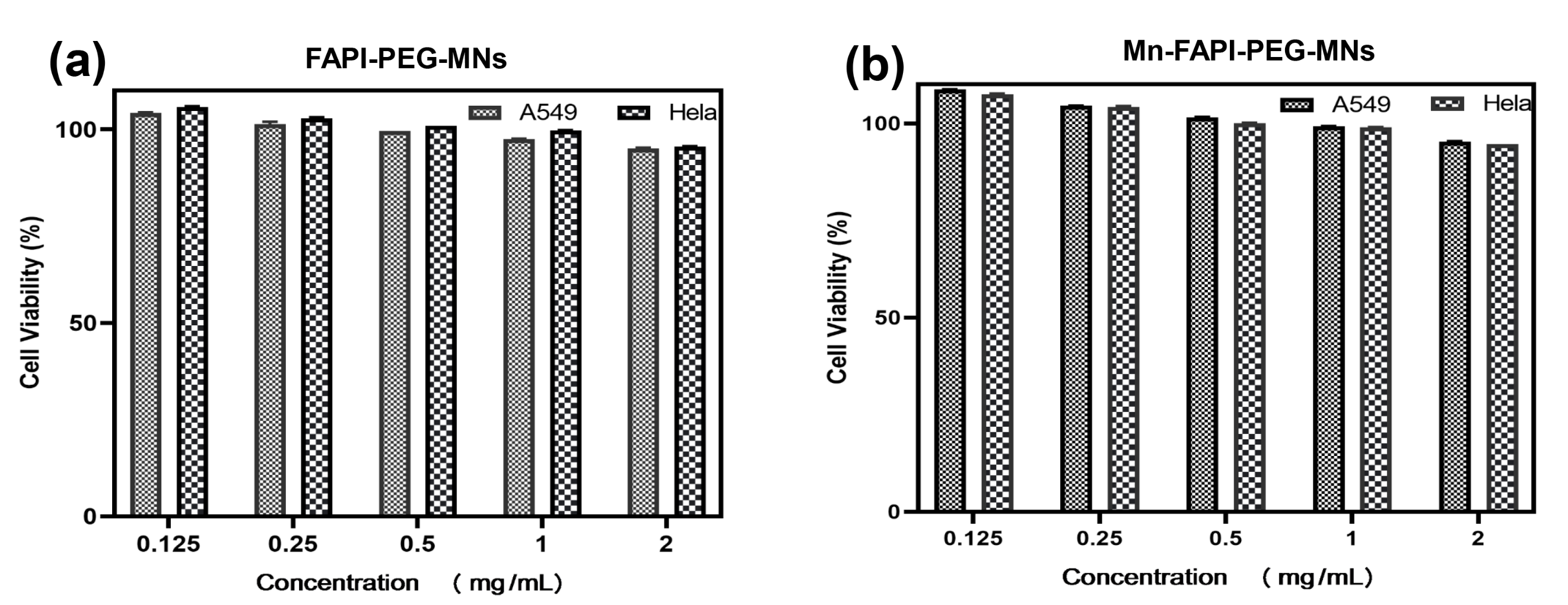


**Figure S8**. MTT assay using a) A549 and b) Hela cells with FAPI-PEG-MNs and Mn-FAPI-PEG-MNs concentrations 0.125, 0.25, 0.5, 1 and 2 mg/mL after 24 h incubation at 37 °C.


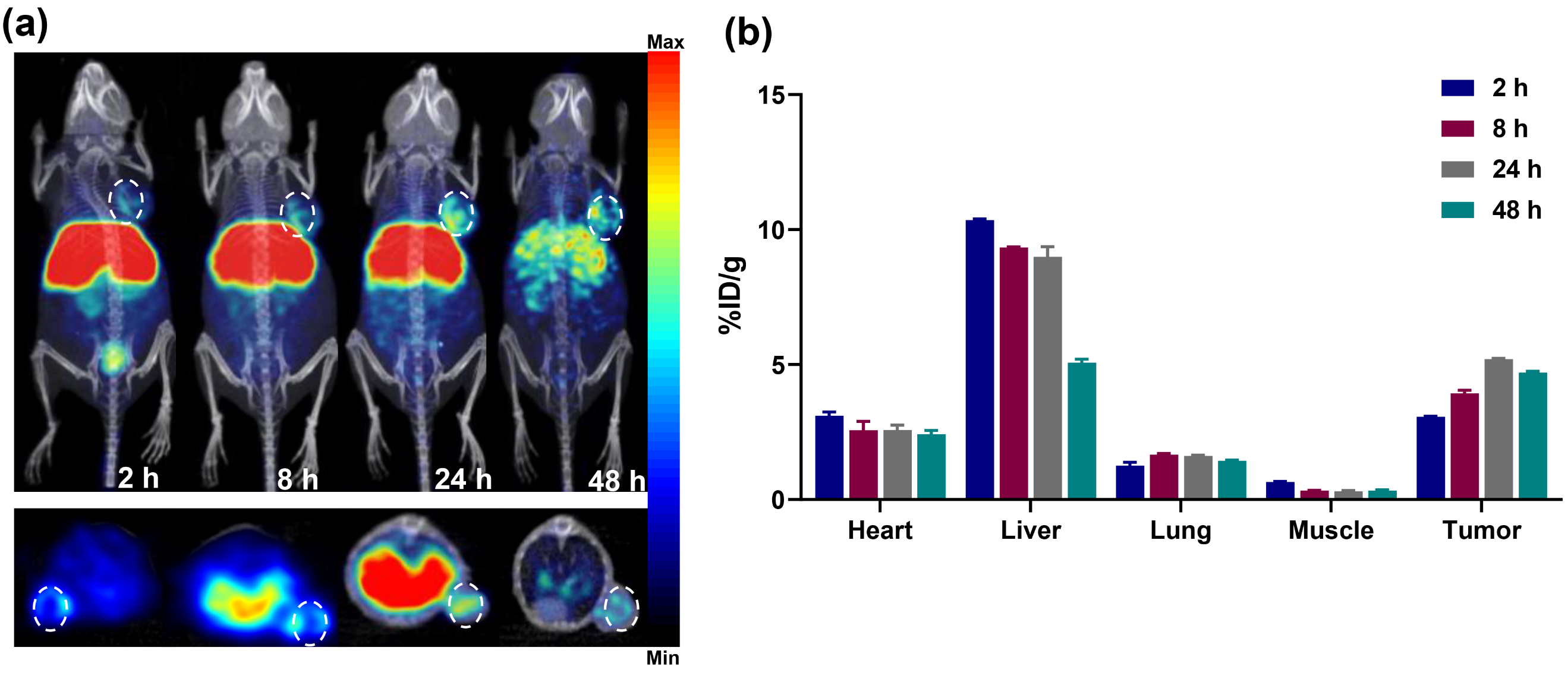


**Figure S9**. a) The Micro-PET/CT images of (^64^Cu, Mn)-FAPI-PEG-MNs in mice bearing U87 MG tumor model at different time points. b) The uptake of (^64^Cu, Mn)-FAPI-PEG-MNs in mice bearing U87 MG tumor model of organs (heart, liver, lung, muscle, and tumor) measured by ROI. Data were presented as %ID. g^−1^.


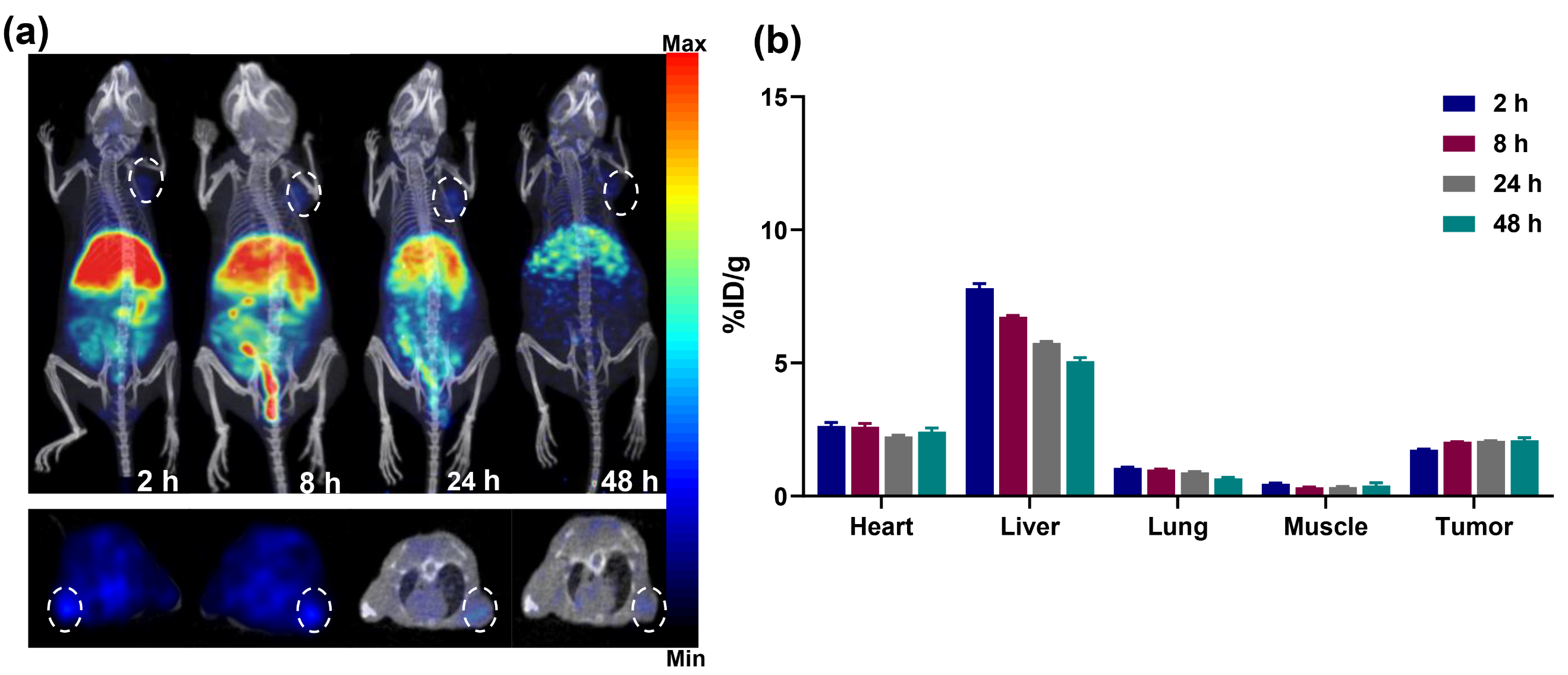


**Figure S10**. a) The Micro-PET/CT images of (^64^Cu, Mn)-FAPI-PEG-MNs in mice bearing A549 tumor model at different time points. b) The uptake of (^64^Cu, Mn)-FAPI-PEG-MNs in mice bearing A549 tumor model of organs (heart, liver, lung, muscle, and tumor) measured by ROI. Data were presented as %ID. g^−1^.


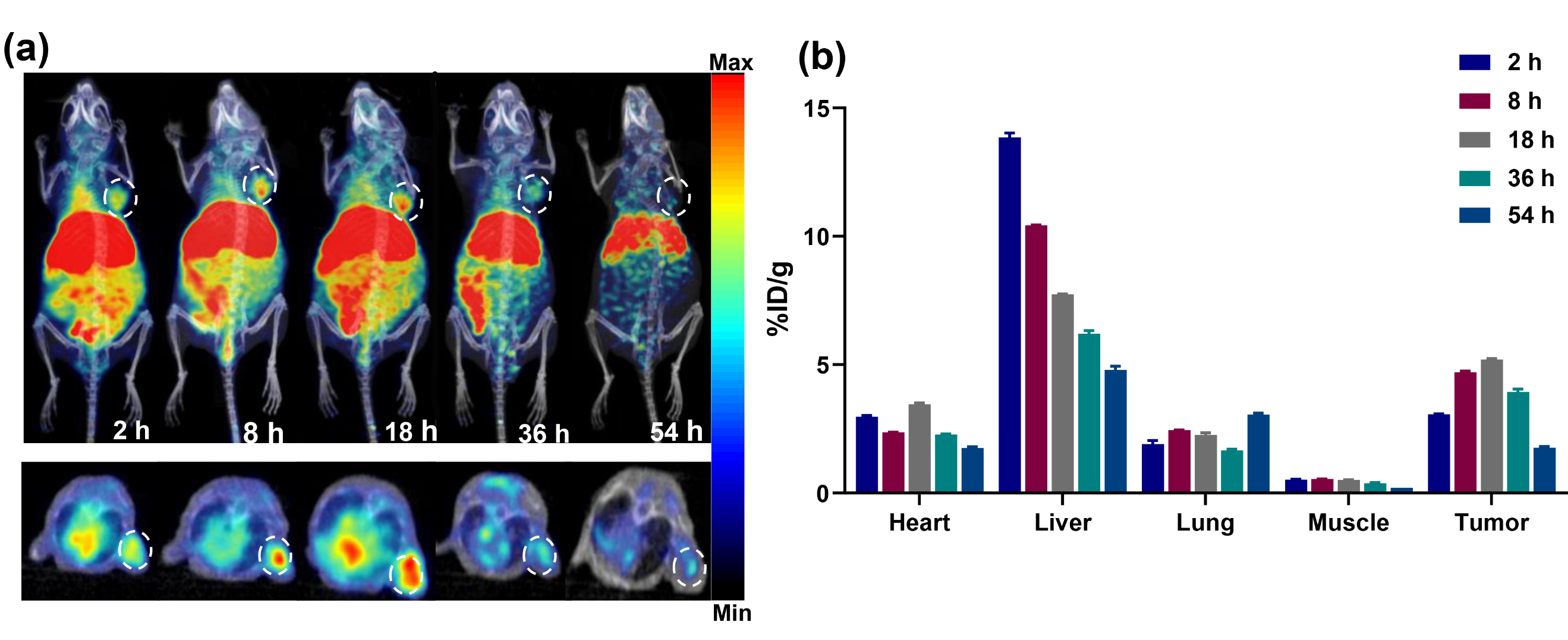


**Figure S11**. a) The Micro-PET/CT images of (^64^Cu, Mn)-FAPI-PEG-MNs in mice bearing U87 MG tumor model at different time points after injecting with hyaluronidase in advance. b) The uptake of (^64^Cu, Mn)-FAPI-PEG-MNs after injecting with hyaluronidase in advance in mice bearing U87 MG tumor model of organs (he1art, liver, lung, muscle, and tumor) measured by ROI. Data were presented as %ID. g^−1^.


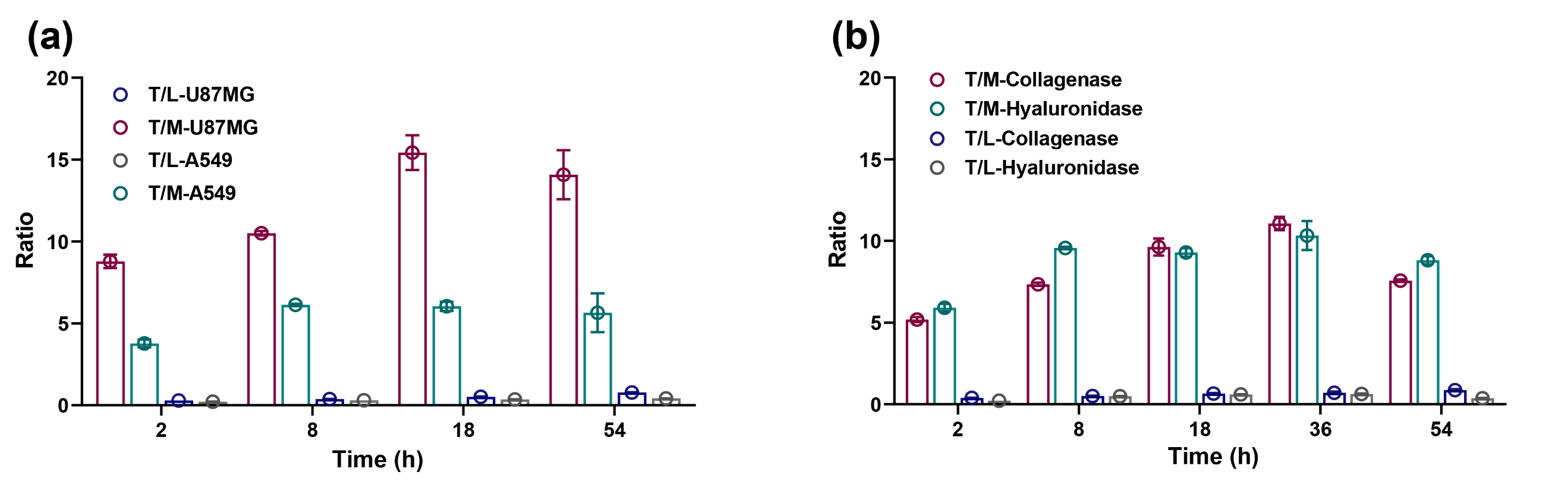


**Figure S12**. a) T/M, T/L ratios (both based on ROI) of (^64^Cu, Mn)-FAPI-PEG-MNs in the U87 MG and A549 tumor model. b) T/M, T/L ratios (both based on ROI) of (^64^Cu, Mn)-FAPI-PEG-MNs in the U87 MG tumor model after injecting with collagenase and hyaluronidase in advance in mice bearing U87 MG tumor model.

1. * L. Wen, C. He, and Y. Guo are co-first authors. [↑](#footnote-ref-1)
2. # **Corresponding author.** *E-mail addresses:* [xialei9012288@126.com](mailto:xialei9012288@126.com) (L Xia), [pekyz@163.com](mailto:pekyz@163.com) (Z Yang), zhuhuananjing@163.com (H Zhu) [↑](#footnote-ref-2)
